# Lead in Air: Adjusting to a New Standard

**DOI:** 10.1289/ehp.118-a76

**Published:** 2010-02

**Authors:** Charles W. Schmidt

**Affiliations:** **Charles W. Schmidt**, MS, an award-winning science writer from Portland, Maine, has written for *Discover Magazine, Science, and Nature Medicine*

When it comes to industrial lead processing, The Doe Run Company’s smelter is in a class by itself. Perched on the banks of the Mississippi River in Herculaneum, Missouri, the smelter’s blast furnaces convert vast amounts of lower-grade ore into more than 125,000 tons of nearly pure commercial-grade lead every year. In operation since 1982, this is both the nation’s largest primary lead smelter and its largest point source for lead emissions, with just over 59 tons of lead released to the air in 2005, according to the most recent figures from the National Emissions Inventory of the U.S. Environmental Protection Agency (EPA). By comparison, that year’s next-highest emitter—a Missouri lead recycling facility also operated by Doe Run—released 12.4 tons.

Doe Run’s smelter in Herculaneum may be the nation’s largest point source for air lead emissions, but it’s not the only one. The National Emissions Inventory, whose next release is expected 31 December 2010, lists 200 facilities emitting between one-half and 1 ton of the metal annually and 139 facilities emitting more than 1 ton. These facilities, which include smelters, battery recyclers, metal foundries, power plants, and airports, represent new and ongoing sources of lead air pollution that will soon draw additional scrutiny from the EPA. Among them, only one—Doe Run’s Herculaneum smelter—put its surrounding community out of compliance under the original National Ambient Air Quality Standard (NAAQS, usually pronounced “nax”) for lead under the Clean Air Act. That will soon change, however, for in 2008, the EPA dropped the lead NAAQS for the first time in 30 years, from 1.5 μg/m^3^ to 0.15 μg/m^3^.

States have until 2017 to meet the new standard. But if 2005–2007 emissions data hold, up to 18 additional locations will be out of attainment with the new lead NAAQS, including communities in Alabama, Colorado, Florida, Illinois, Indiana, Minnesota, Missouri, New Jersey, Ohio, Pennsylvania, Tennessee, and Texas. Meanwhile, the emergence of new nonattainment areas puts a spotlight on point sources, or identifiable sources of concentrated emissions, which—in the EPA’s view—account for the dominant share of lead air risks in the United States today. “It’s appropriate to infer that point sources—especially now with the removal of lead from gas—are the main routes of exposure to lead in outdoor air, at least in this country,” says Lewis Weinstock, group leader of the EPA Ambient Air Monitoring Group.

That view puts the EPA squarely at odds with the lead industry. David Weinberg, a lawyer with Battery Council International (BCI), a trade group in Washington, DC, argues that “legacy” contamination from old leaded gas and house paint contributes more to elevated blood lead than point-source emissions do. Exposure to legacy lead occurs both by ingesting contaminated soils and by inhaling resuspended road dusts and soils. “We’re making tremendous strides controlling industrial lead emissions with closed-loop cycles,” Weinberg says. “And as you lower ambient levels of concern, historic sources—i.e., residual paint and gas contamination—become increasingly important.”

Philip J. Landrigan, a pediatrician and lead researcher at Mount Sinai School of Medicine, points out, however, that the dominant source of exposure for a particular child depends on where the child lives. “It is very important to document lead emissions from *all* of these sources,” he says, “because even the smallest exposures to lead are now understood to cause damage to the developing brains of young children.”

## Beefing Up the Network

It’s impossible to know all the locations that will be put out of attainment under the new standard. That’s because in many parts of the country, the lead monitoring network isn’t developed enough to measure compliance with it, Weinstock says.

During the network’s peak activity, in 1980, more than 900 monitors were positioned near point sources, along roadsides, and in urban locations. Like large vacuum cleaners, the samplers used in the monitoring network trap airborne lead in filters, which are then removed and analyzed. Readings are taken every 6 days, with ambient air levels calculated as rolling 3-month averages, yielding 12 averages per year (under the old NAAQS, averages were calculated quarterly, yielding 4 averages per year). After the phaseout of leaded gas began in 1976, the amount of lead in air fell sharply, Weinstock says, and the EPA reduced its monitoring network accordingly. By 1998, the number of active monitors had dwindled to 290, and today roughly 130 are operational, mostly near point sources, according to agency spokeswoman Cathy Milbourn.

Now the EPA proposes to expand its network with new monitors placed near point sources to assess NAAQS compliance, and also at the agency’s 80 NCore stations, which monitor multiple airborne pollutant levels for research rather than regulatory purposes. If approved, the new monitors would begin operating in 2011. As part of this new strategy, the EPA also proposes to rescind an earlier decision to phase in 100 monitors in cities with populations of more than 500,000 people. Details of the proposed new monitoring strategy were described in the 30 December 2009 *Federal Register*.

This change in emphasis reflects declines in childhood blood lead coinciding with the reduction in leaded gas. National surveillance data collected by the Centers for Disease Control and Prevention and published on its Lead website (http://www.cdc.gov/nceh/lead/) show that in 1998, nearly 3.5 million children had blood lead levels exceeding 10 μg/dL (the action level at which intervention is recommended), compared with about 250,000 today.

Determining which sources to monitor hasn’t come easily. After an extensive deliberation, described in the 3 March 2008 EPA memorandum “Lead NAAQS Ambient Air Monitoring Network: Network Design Options Under Consideration,” Weinstock and his staff concluded the EPA should focus on sources emitting at least a half-ton of lead per year. The EPA’s decision to set the value at 1 ton drew an immediate outcry from the Missouri Coalition for the Environment Foundation, the Natural Resources Defense Council, the Coalition to End Childhood Lead Poisoning, and Physicians for Social Responsibility. Arguing that the decision to go with a 1-ton threshold was arbitrary and capricious, these organizations petitioned the EPA to reconsider, and in December 2009 the agency relented, reproposing the originally recommended half-ton threshold.

Avinash Kar, a staff attorney with the Natural Resources Defense Council, emphasizes that just because a facility emits half a ton of lead doesn’t mean restrictions on the facility are imminent. Instead, the goal of monitoring is to gather more information in light of the risk that such facilities may lead to NAAQS violations. Indeed, the relationship between point-source emissions and air levels depends on many factors, says Armistead Russell, a professor of environmental engineering at the Georgia Institute of Technology and member of the EPA Science Advisory Board. Among them are smokestack height, distance to property fence lines, size of the particles emitted, and windspeed. These factors are incorporated into dispersion models that pinpoint where the highest air concentrations will likely occur. The influence of local topography and meteorology can exert more influence on maximum air concentrations than how much lead comes out the stack, Russell adds.

With its new strategy, the EPA will take a closer look at how much point sources contribute to exposure. So far, the clearest linkage appears to exist in Herculaneum. A 2001 health consultation report released by the Missouri Department of Health and Senior Services (MDHSS) revealed that 28% of 118 young children tested had blood lead levels exceeding 10 μg/dL—far higher than the national average for that year of 7.6% and the Missouri state average of 8%. Among children living closest to the plant, 45% had blood lead exceeding 10 μg/dL. The most recent data from the MDHSS, for 2006–2008, showed no evidence of blood lead levels above 10 μg/dL in any child tested in Herculaneum—including those living in high-risk areas.

Multiple factors account for this change, says MDHSS environmental specialist Jonathan Garoutte. Prodded by federal, state and local agencies, as well as the community itself—Doe Run bought out properties within a half-mile radius of the plant, redirected truck traffic away from local neighborhoods, and remediated yards where lead levels of up to 33,100 ppm had once been detected. Moreover, Doe Run spokeswoman Tammy Stankey says that with process controls annual lead emissions from the smelter fell to 22.4 tons in 2008 (a 62% reduction over 2005 emissions), as reported to the EPA Toxics Release Inventory.

Yet in October 2009, the EPA reported that more than a third of 372 soil samples taken within a mile of the smelter were contaminated with lead at levels above the agency’s 400-ppm threshold for remediating play areas. Most of those properties had already undergone EPA-ordered lead remediation during the last decade, but “[w]hile Doe Run has taken some steps in recent years to reduce lead emissions, those efforts clearly fall short of what was necessary,” said acting EPA regional administrator William Rice in a 26 October 2009 press release.

## Point Sources versus Legacy

Relationships between point-source emissions and exposure aren’t always obvious, and it can be challenging to directly connect them to elevated blood lead in children. The new lead NAAQS was calculated with models that relate what’s in the air to what ends up in children’s blood by way of their exposures to soil, house dust, and other media. When the first NAAQS was derived in 1978, leaded gas was ubiquitous, and the EPA determined that most exposure to the toxicant came from breathing exhaust fumes. Today, however, in developed countries that don’t use leaded gas, the dominant exposure route to lead air emissions has shifted to incidental ingestion of lead particles that drift to the ground.

In many places, point sources merely add to legacy contamination, and it can be difficult to distinguish this new fraction from what was already there, according to Howard Mielke, a professor at the Tulane University/Xavier Center for Bioenvironmental Research. Scientists hope to “fingerprint” lead isotopes to identify originating sources of lead pollution, Mielke says, but these research efforts are still preliminary.

Industry groups, meanwhile, insist that most lead-poisoned children live in poor, urban neighborhoods where legacy sources account for the greatest risk. In a 4 August 2008 letter to the EPA in response to the agency’s proposal to strengthen the lead NAAQS, Timothy J. Lafond, chair of BCI’s Environment Committee, argued it’s pointless to go after industrial sources when lead threats to children occur mainly in high-poverty areas. Quoting a technical report supplied on contract to BCI by the Menlo Park, California–based consulting firm Exponent, he wrote, “‘there is practically no relationship between the emission rate and maximum monthly lead concentrations’ at sites other than the Herculaneum smelter.” The better approach, Lafond wrote, would be to concentrate air monitors in high-poverty areas, which he says could be easily located with census data.

Legacy lead can remain in soil for hundreds of years, posing ongoing threats to children, Mielke acknowledges. And those contaminated soils can be resuspended in the air, especially during hot summer months, he says, posing threats that cause blood lead levels to vary on a climatic basis. Mielke has analyzed data from the Louisiana Childhood Lead Poisoning Prevention Program in conjunction with high-resolution soil sampling by census tracts. “We tend to see levels in soil from poor, inner-city neighborhoods that range between 500 and 1,000 ppm,” he says. “And those are the levels that seem to correlate with blood lead concentrations beyond 10 μg/dL.”

Marie Lynn Miranda, director of the Children’s Environmental Health Initiative at Duke University, adds that lead risks to the urban poor generally occur where homes were built before the regulations phasing out leaded gas and house paint. And in general, she says, the older urban neighborhoods tend to be found in northern regions of the United States. However, she adds, there are exceptions, New Orleans among them.

Mielke’s research shows that leaded gas is still a surprisingly important source of urban contamination. “You can calculate how much lead would have been generated in a half-mile radius around a major intersection with a hundred thousand cars per day, and it’s like having a secondary smelter in there,” he says.

All this suggests that at least in urban locations—and particularly during the late summer—inhalation could play a greater role in exposure than might otherwise be assumed. But that assumption brings up other uncertainties, Mielke says, such as differences relating to ingestion and inhalation among younger and older children, who might not engage in as much hand-to-mouth activity. “It’s very complex, and sadly, we’re using children to figure all this out,” Mielke says.

Given the threats posed by legacy contamination, Weinberg stresses the EPA and state agencies should focus on areas where lead risks are highest, rather than targeting point-source emissions. Indeed, says Kar, “Legacy pollution can and is being addressed under different programs such as lead hazard reduction programs and Superfund, in addition to the Clean Air Act. It’s just not being addressed enough.”

Kar points out the EPA’s regulatory mandate under the Clean Air Act is primarily focused on targeting sources of new pollution, although the act does consider legacy contamination that contributes to future violations of air pollution standards—for example, resuspended soil dust at a lead-polluted site. “The contribution of such resuspension is not excluded when violations of the NAAQS are determined, and strategies may need to be developed to prevent such ambient air pollution,” he says.

Weinstock emphasizes that companies can try to avoid point-source monitoring with their own dispersion models. If those models show air levels won’t exceed 50% of the lead NAAQS, he says, monitoring requirements can be waived by the responsible agency (usually a state agency), but only with the EPA’s permission.

The bright spot in all this is that on average, blood lead levels appear to be falling nationwide. Yet that benefit isn’t always shared equitably with inner-city children, who still bear the brunt of ongoing lead pollution. Moreover, when one considers how small a dose lead is believed to harm a child’s brain—evidence now suggesting that in fact there is no safe threshold of exposure—emissions measured by the ton should give anyone pause.

## Figures and Tables

**Figure:**
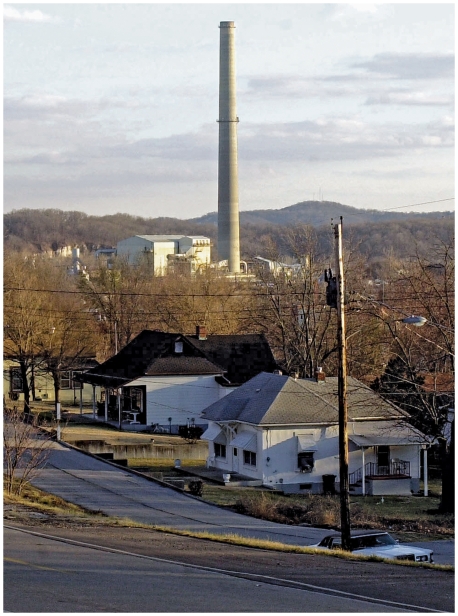


**Figure:**
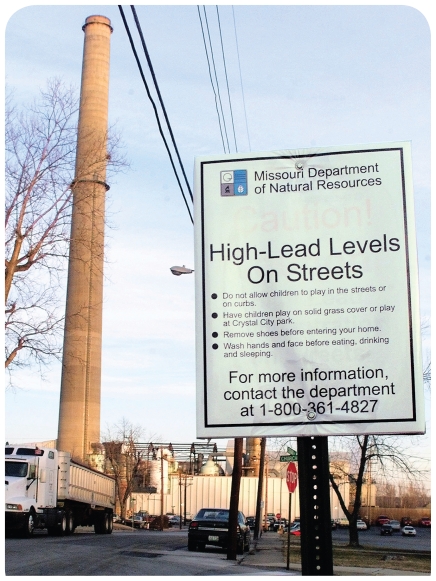
**Opposite:** The smoke stack of the Doe Run lead smelter rises behind the town of Herculaneum, Missouri. **Above:** A sign warning of dangerous lead levels is posted near the Doe Run lead smelter, 15 January 2002. In 2007 the EPA ordered Doe Run to wash its trucks before leaving the smelter grounds and clean the streets regularly to help prevent lead contamination. By October 2009, however, new test results showed Herculaneum soil was still being contaminated.

**Figure:**
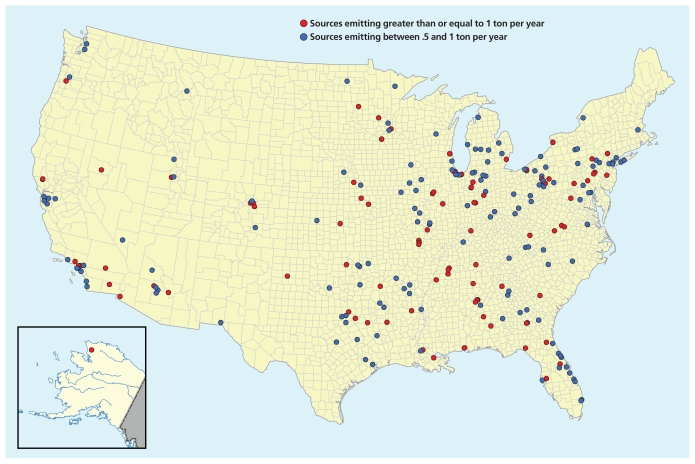
Sites for Potential Monitoring **Data from the 2005 National Emissions Inventory reveal sites that emit at least a half-ton of lead into the air each year. If policy changes proposed by the EPA in December 2009 are approved, such sites will become subject to monitoring to ensure NAAQS compliance**. Source: U.S. Environmental Protection Agency National Emissions Inventory 2005. Adapted by Matthew Ray/EHP.

